# The lived experience of patients with obesity at a metropolitan public health setting

**DOI:** 10.1186/s12913-022-08928-w

**Published:** 2022-12-16

**Authors:** Fiona M. Pazsa, Catherine M. Said, Kimberley J. Haines, Eloise Silburn, Melina Shackell, Danielle Hitch

**Affiliations:** 1 Physiotherapy, Western Health, Melbourne, Victoria Australia; 2 Physiotherapy, The University of Melbourne, Melbourne, Victoria Australia; 3 Australian Institute for Musculoskeletal Science, St Albans, Victoria Australia; 4 Department of Psychology, Western Health, Melbourne, Victoria Australia; 5 Allied Health, Western Health, Melbourne, Victoria Australia; 6 Occupational Therapy, Deakin University, Geelong, Victoria Australia

**Keywords:** Obesity, Inpatient, Patient experience, Hospitalisation, Models of care, Qualitative, Patient perspective, Bariatric, Patient centered care

## Abstract

**Background:**

Patient-centred care models for acutely hospitalised people living with obesity are poorly understood and the quality of evidence low.

**Objective:**

The aim of this study was to explore and better understand the lived experience of people living with obesity, in the inpatient hospital context.

**Design:**

A qualitative methodology using Interpretative Phenomenological Analysis (IPA) was used. Data were collected via a single semi-structured interview with each participant.

**Setting and participants:**

The study was completed at a metropolitan public health service. Ten previously hospitalised patients who live with obesity were included.

**Results:**

Three main themes emerged: meeting physical care needs of people with obesity on hospital wards, interpersonal interactions between patients and healthcare professionals, and the psychosocial impact of being obese in the hospital setting. Priorities included timely provision of appropriate equipment and infrastructure design to meet care needs and facilitate better wellbeing. To improve patient experience, an emphasis on basic principles of quality care provision to enhance interpersonal interactions, along with improved awareness of the impact of weight bias and obesity stigma in healthcare are supported. Participants found hospitalisation stressful, but valued support from healthcare professionals regarding weight loss.

**Discussion:**

These data provide new insights in to the lived experience of people living with obesity in the hospital setting. Items which are low cost, such as appropriately sized chairs and gowns, as well facilitators to independent mobility such as electric wheelchairs are suggested to improve both experience and care outcomes. Interpersonal interactions demonstrated obesity stigma in the hospital setting, with participants expressing the desire for more appropriate communication. People living with obesity self-reflected in the inpatient setting, suggesting that staff should be trained to utilise the opportunity to provide weight loss advice.

**Conclusions:**

The themes identified in this study provide insight into the lived experience of people with obesity in hospital. This understanding provides direction for the development of improved models of care for people living with obesity in this setting and beyond.

**Supplementary Information:**

The online version contains supplementary material available at 10.1186/s12913-022-08928-w.

## Introduction

The rising prevalence of obesity, defined as abnormal or excessive fat accumulation that presents a risk to health is a key challenge for healthcare systems internationally [1]. Obesity is measured using the body mass index (BMI) (weight in kilograms divided by the square of height in metres), with a BMI of 30 or more considered obese [1]. Obesity is a risk factor for multiple diseases, and is associated with increased hospitalisation rates and healthcare costs [2]. People with obesity require a specialised care approach during hospitalisation due to their size, body habitus and compounding comorbidities. Although the effects of obesity have been well documented in terms of morbidity and mortality, less is known about what it is like to live with this complex and chronic disease [3], particularly in the context of being in hospital. Previous research has demonstrated that obesity stigma and weight bias occur in the general community generally [3], and in the hospital setting [4]. Navigating such complexities can negatively impact on a person’s care experience. In addition, there is limited understanding of important factors in care delivery while in hospital for people living with obesity. This makes it difficult to develop patient-centred, effective models of care which meet the needs of people living with obesity in hospital.

Positive patient experiences are known to be associated with improved patient safety and clinical effectiveness [5]. Conversely, negative patient experiences can lead to avoidance of care, mistrust of healthcare professionals and poor engagement in treatment for patients with obesity [4]. There is also strong evidence that people with obesity have poorer health outcomes in a range of patient groups [6–9]. This is attributable to the physiological impact of being obese and subsequent the lack of quality of care. This increased risk of suboptimal outcomes and lack of understanding makes it difficult to identify the most important factors for delivery of safe, effective, and high-quality care for these patients.

Most of the current literature regarding care of people living with obesity while in hospital is based on clinical opinion rather than being informed by lived experience or scientific evidence. The aim of this study was to bridge this gap by exploring the lived experience of people with obesity admitted to hospital. The findings will inform future service improvement for people with obesity requiring hospital-based care related to any aspect of their health.

## Methodology

This study utilised a qualitative methodology and employed Interpretative Phenomenological Analysis (IPA) to explore the research question. IPA aims to explore ‘lived experience’ and how people make sense of it, via first person subjective accounts [10]. It is based on the assumption that human beings are “constantly engaged in the process of interpreting their experiences and such interpretations are necessary for them to reach an understanding of the events or experiences that mark their life” [11]. In healthcare, the lived service experience of consumers can be of great value in driving service improvement that is relevant to their needs, and inclusive of their perspectives.

### Setting

This study was conducted at a tertiary public health service in metropolitan Melbourne, Australia. The health service has three acute campuses and provides outpatient and community services. The local population cared for by this health service is characterised by socioeconomic disadvantage, and higher than average prevalence of obesity [12] .

The health service has initiated service innovations to facilitate best care in people with obesity in recent years. The Bariatric Assessment Team (BAT) was established by a Bariatric Working Party to address the need to improve care for inpatients living with obesity. The BAT is a referral-based service comprised of a dedicated multidisciplinary team including Occupational Health and Safety (OHS), Physiotherapy, Occupational Therapy and Nursing. Adults (aged 18+) can be referred to BAT if they have a BMI greater than or equal to 40 AND their weight exceeds (or appears to exceed) the safe working load/weight capacity of standard hospital equipment OR their size restricts the use of standard furniture, mobility or functional level. The BAT provides recommendations and facilitates education around equipment provision, bed allocation, manual handling, skin integrity, referrals, and discharge planning.

### Participant recruitment

Purposive sampling was utilised to identify adults with obesity who had an inpatient admission at the organisation between March 2018 and July 2019, as identified by referrals to the Bariatric Assessment Team (*n* = 84). Potential participants were invited to participate via a letter, which was followed up by telephone contact (from a member of the research team were not known to the participant). Those who were unable or unwilling to provide written consent; had a language or cognitive impairment or medical status which impacted participation; or were unable to attend a face-to-face interview were excluded. Where appropriate, an interview was scheduled, and explicit written consent obtained prior to commencement. Participants were able to have a carer present if they wished to, with two choosing to do so. A professional interpreting service was available for participants for whom English was not their first language, which was used for one interview. During the interviews, researchers and participants were aware that if a participant did not wish to answer a question, it may be skipped or if preferable the interview ceased completely. Should a participant become upset or distressed as a result of participation in the research project, the research team would arrange for counselling or other appropriate support. This was not required for any participant.

The number of interviews required to be completed, was ascertained by the adequacy of the data (considering richness and complexity) to address the research question [13]. The size of the sample was determined by the model of “information power” [14]. The more information the sample holds, relevant to the research question, the lower the number of participants required. No further interviews were conducted once information redundancy was reached, meaning no new codes or themes emerged from the data.

Ten participants consented and were interviewed. Of the 84 people with obesity invited by letter to participate, 24.1% could not be contacted by phone. After phone contact, 30.7% did not consent to participate, 16.4% had precluding medical conditions or were deceased, and 6.5% were excluded due to cognitive impairment. Three participants gave verbal consent on the telephone but were unable to attend or did not attend an interview.

### Data collection

Data were collected using face-to-face semi structured interviews with a specially designed interview schedule. The design of the schedule was guided by previous research into non-stigmatizing language for people with obesity [15–17] (see Additional file 1: Appendix A) and reviewed by members of the research team with experience in qualitative research. The interviews were conducted by authors DH and ES, who had no previous clinical contact with participants. Interviews took place at the organisation and had a duration of 30 to 60 minutes, with a total of 5 hours and 36 minutes of recording captured. Careful consideration was given to sourcing appropriate interview venues, with accessible rooms and appropriately sized furniture. Interviews were audio recorded and transcribed verbatim by an external provider. Participant identity was protected through this process; names were not used during the interview and transcripts were de-identified. Following transcription, a copy of the interview was sent to the participant for member checking. No amendments were made to transcripts by participants during this process.

### Data analysis

Dedoose software was used to manage and analyse the data [18]. Each transcript was first independently subjected to thematic analysis by two researchers (FP, TJ, or TH). The researchers produced codes for the interpreted meaning of each passage. A reflexive thematic analysis style was adopted, with the codes considered fluid and open to amendment to “fully embrace qualitative research values and the subjective skills the researcher brings to the process” [19]. As per Table 1, a list of overall themes was then formed collaboratively from the subthemes identified by all members of the research team by comparing and combining codes from each participant, and then between the patients. Subthemes that were deemed to be more general were further analysed and data was allocated to an overall theme at the agreement of the research team. The data comprising the overall themes key themes were summarised, to provide a detailed account of common aspects of lived experience from the patient perspective. Finally, the findings were used for interrogation with and comparison to existing research [10].

**Table 1:** Data Analysis Mapping

Overall Theme	Subthemes Included
Meeting Physical Care Needs	Equipment Environment Manual Handling Delays in Care Transitions Between Wards / Discharge Relevant Negative Service Experience Relevant Positive Experiences Relevant Suggestions for Improvement
Interpersonal Interactions	Communication Interpersonal Interactions Relationship With Staff Relevant Negative Service Experience Relevant Positive Experiences Relevant Suggestions for Improvement
Psychosocial Impact	Carers / Family Weight Loss Self Reflection Emotional Impact Loss of Independence Relevant Negative Service Experience Relevant Positive Experiences Relevant Suggestions for Improvement

### Trustworthiness

The four aspects of trustworthiness (credibility, transferability, dependability, confirmability) in Guba’s Model of Trustworthiness of Qualitative Research were referenced to ensure the quality of the study [20]. Credibility was achieved through the following methods. Reflexivity was enacted by researchers as statements of the researchers perspectives on obesity and The Attitudes Toward Obese Persons Scale (ATOP) [21] were completed and revisited throughout the study. This process supported researchers in critically reflecting on their position in the research team and how their stance is taken into account. As stated previously, participants were also offered the opportunity to member check their transcripts. The use of multiple investigators to verify results also adds credibility. A diverse sample was sought to be representative of the clinical population, which enhances (but does not guarantee) transferability. Dependability was supported by the inclusion of detailed descriptions of research methods and multiple researchers collectively interpreting the data.

## Results

The majority of participants were men (*n* = 6), and the mean age was 51 years (± 14, 25 – 73). The mean BMI (57.4) met the criteria for morbid obesity (greater than or equal to 40) [22] with the mean weight of participants was 158 kg (± 25.8, 109 - 194). The mean length of hospital admission for participants was 29.6 days, which exceeds the average length of stay of 5.3 days for emergency admissions to Australian public hospitals [23]. Reasons for admission were not specifically related to obesity, with the most common reason being complex orthopaedic issues (*n* = 3), followed by lower limb cellulitis (*n* = 2). Participant characteristics are provided in Table 2.

**Table 2:** Participant Characteristics

Participant	Gender	Age (years)	Language	BMI	Weight (kg)	Length of admission (days)	Admission Reason
1	Male	50	English	51.9	168	5	Complex orthopaedic issues
2	Male	41	English	58	194	2	Lower limb cellulitis
3	Female	36	English	82.8	179	39	Complex orthopaedic issues
4	Female	51	English	50	109	7	Abdominal issues
5	Female	59	Arabic	Unable to obtain	129	44	Stroke
6	Male	73	English	Unable to obtain	164	16	Cardiac issues
7	Male	25	English	57.4	170	129	Polypharmacy overdose
8	Male	62	English	35.3	127	6	Stroke
9	Male	44	English	53.1	162	19	Lower limb cellulitis
10	Female	67	English	70.8	179	29	Complex orthopaedic issues

Three themes were identified within the data: 1) meeting physical care needs of people with obesity on hospital wards, 2) interpersonal interactions between patients and healthcare professionals, and 3) the psychosocial impact of living with obesity in the hospital setting. Notably, all participants initially indicated that their overall service experience was positive but were able to describe and elaborate on negative or challenging aspects as the discussion wore on, highlighting the benefit of in depth conversation.

### 1. Meeting physical care needs

Codes related to this theme were prevalent within the data, and generally reflective of negative experiences. Lack of timely provision of appropriate equipment and unsuitable infrastructure for people with obesity was reported by half the participants (*n* = 5): “*It was a bit hard for them to find the things that I did need*.” (Participant 10). This had a significant impact on their experience, resulting in poor outcomes including increased pain and dependence: “*That’s all I can do because I don’t have anywhere comfortable to sit. Just laying in the bed and I get a sore back*” (Participant 7). The persistent nature of this issue also led to other bad experiences, such as disagreements with staff: *“I had an argument with her, because they made me sleep in a chair, a chair like that, all night, because they reckoned they couldn’t find a mattress”* (Participant 2). Some participants also recounted incidents where they experienced a loss of personal dignity, due to inappropriate equipment or environments. These situations prompted them to attempt to resolve the issues for themselves: “*Yeah, you’ve got to have a gown on, they don’t fit, what do you want me to do? So I put my face washer over, you know…”* (Participant 7).

Others perceived they were the cause of harder or more burdensome work for the staff due to their size: “*I think they just looked at me thinking that she’s too big … too much work sort of thing*” (Participant 5). For one participant, the cumulative impact of these negative experiences resulted in them choosing to leave hospital and cease treatment: “*I was at [hospital] first and I ended up checking myself out because basically the rooms were tiny. Built for someone that’s not even half my size. Small bed, small everything, small bathroom, couldn’t get around. The toilet, I’m sad to say I broke it.*” (Participant 7).

Resources commonly perceived as problematic included equipment necessary for basic care provision, including beds, chairs, assistive equipment (i.e. frames, commodes), hospital gowns, and continence aids. Without prompting, half the participants identified a lack of access to independently operated electric wheelchairs as a significant barrier to mobility and participation, impeding wellbeing: *“if I said look can I get a wheelchair for 2 minutes and I can drive myself down to the bloody cafeteria, have a coffee and then come back up I’d feel 100%. I’d be out of the room”* (Participant 6).

Conversely, when suitable equipment was provided to meet their basic care needs, more positive descriptive language such as “*good*” and “*comfortable*” was used. These descriptors most often referred to equipment that accommodated their size and was available when required.

Finally, multiple participants expressed a preference for a particular site within the organisation, which has newer infrastructure, and a limited number of rooms designed and furnished to meet the needs of people living with obesity. In contrast, the patient experience at the health service site with older infrastructure was much less positive, as that environment posed far more barriers to care.

Table 3 provides additional participant quotes for this theme.

**Table 3:** Additional Participant Quotes

Theme	Participant Quotes
Meeting Physical Care Needs	*“Yeah, I mean I see normal people sit in the chair and it’s okay, but when I sit in the chair it feels very small and because I’m so big. And it’s very hard to move and …* “(Participant 9) *“Yeah I can’t move my legs and I can’t get up out of the chair properly, and I can’t walk and all this. But the staff that’s in there they want me to do, move here, do this, do that. As I tried to point out that if I … they want me to sit up very far but I tell them I can’t I have to take my time.* “(Participant 9) *“Yeah, but that’s the only thing, because they didn’t have one big enough”* (Participant 10) *“Yeah. And they said, sorry, you can’t have a shower because the bathroom’s too small*.” (Participant 10)
Interpersonal Interactions	*“they dropped tablets and picked them up off the floor and try to put them into your mouth is, yeah, it’s a bad thing.”* (Participant 2). *“in the hospital I fell over once… I called the people to come. But no one came.”* (Participant 9). *“I just want a bit more communication”* (Participant 9).
Psychosocial Impact	*“So they came and they did an assessment and they had an interpreter and they kept actually bringing a psychologist, they used to come every week or so and do all these different assessments to make sure that mentally I’m good.”* (Participant 5) *“Because you’re worried, you know, it’s your health; you don’t know what’s happening.”* (Participant 3)

### 2. Interpersonal interactions

Whilst participants recollected both negative and positive interpersonal interactions, only negative experiences were described in clear and specific language. Not all negative experiences recounted were weight related, suggesting the experiences described were not necessarily unique to patients living with obesity.

Most participants described adverse experiences, where they perceived disinterest in their care and a lack of compassion: *“they don’t really care, like do they need a break from the job to start liking it again?”* (Participant 2). Many participants described a lack of empathetic communication from staff members, included the feeling of being ignored: *“I’ve had other nurses arguing while they’re drawing needles up over me, and that scared me a bit … when you’re drawing needles up, you should be concentrating, not arguing with each other over a patient”* (Participant 2). In some interactions, participants felt a lack of respect toward them as individuals and as patients: “*I think she could have came in and actually sat down and talked ... but she was just a bit rude”* (Participant 8). In a specific example of obesity stigma, participants reflected on the impact a lack of communication had on them emotionally: *“and how to be able to talk to someone when there’s an obesity problem yeah be able to communicate with them really well and just not look up and down at them, it’s very sad when they do that.”* (Participant 5). A desire for increased communication was also noted: *“…like I said* [staff] *are set in their ways. And they have one way, their way of doing things and they will try to do it their way before listening to what you want.”* (Participant 9). In contrast, participants also described positive care experiences that brought them joy, such as sharing a laugh with staff: *“They were fantastic like they would always come and joke with me. They would laugh which was good, I wanted that smile.”* (Participant 5). Mutual respect and feeling a part of their care team were also valued and appreciated: *“I would respect them; they would respect me …”* (Participant 1). Overall, positive approaches to social communication (such as being diligent, caring and happy and engaging in active listening) underpin many of the descriptions of positive interpersonal interactions.

Table 3 provides additional participant quotes for this theme.

### 3. Psychosocial impact

Psychosocial is defined in this study as pertaining to the influence of social factors on an individual’s mind or behaviour [24]. Participants in this study described challenging and confronting social stressors and psychological consequences during their experience of hospitalisation: *“Yeah it’s not easy when you’re an obese person (sic)… it’s just so horrible because it feels degrading, when we… need to come into hospital.”* (Participant 5). In addition to the lived experience of being in hospital feeling “degrading”, participants perceived themselves as a burden on staff: *“… because I see 2 or 3 or even 4 nurses helping me move,. And it makes me feel bad, no good.”* (Participant 9). Feelings of being stressed at delays in care, or having to wait for usual carers to meet gaps in care were also reported: *“I just had to wait for my wife to come in to try and give me hand afterwards. She was about 2 hours away so I just persevered, but yeah… that sort of stresses me out a bit”* (Participant 7).

The hospitalisation prompted some participants to reflect on their weight: *“Yeah and the way I’m now, … and I don’t like the way I am.”* (Participant 9) and its potential impact on their health and function: *“I used to like walk two hours a day when I used to work and I used to get public transport, so I was very active and then all of a sudden I just became inactive and I started putting on weight… that is upsetting when I look at myself and seeing that I am overweight.”* (Participant 5). Of note, four participants described positive experiences and identified hospitalisation as an opportunity to address their weight issues. Receiving assistance or support with weight loss during admission was highlighted: *“Yeah they were sending me all this food that was good for me. They were fantastic.”* (Participant 5).

Table 3 provides additional participant quotes for this theme.

## Discussion

The findings of this study captured lived experiences of people living with obesity during hospitalisation and provide critical new insights into their experiences. Our findings identified that the key themes for people living with obesity on hospital wards were meeting physical care needs, interpersonal interactions with healthcare professionals, and the psychosocial impact of being obese in the hospital setting. These data should be used to inform innovative care models, given the identified differences in priorities and perspectives between healthcare professionals and patients living with obesity [25]. The themes identified provide direction to drive service improvements in this patient cohort, who experience higher than average length of stay and currently report service experiences which are inappropriate to their needs and potentially damaging to their wellbeing.

A key priority for people living with obesity in hospital was timely provision of equipment to meet their personal needs, which has also been found in previous studies [26]. Planned approaches to ward transfers and systems to facilitate timely sourcing of equipment are needed to streamline this process and may require internal stock reviews or ready access to external suppliers. Previous research has also found patient comfort is compromised when specialised equipment is designed simply as scaled up versions of standard equipment [27]. Larger equipment is also often more expensive; however, the findings here emphasise the potential impact low-cost items (including simple assistive technology, gowns, and continence aids) can have on patient experience. Lack of adequate facilitators for mobility was another common experience, which may also contribute to negative experiences and poorer health outcomes. For example, delays to being able to sit out of bed in a suitable chair is in opposition to best practice inpatient care and increases the risk of hospital associated deconditioning [28]. This barrier also exacerbated a perceived loss of independence, causing a negative impact on health and wellbeing. The often-repeated recommendation of providing access to independently propelled electric wheelchairs reflects a desire by patients to self-manage their participation in meaningful activities whilst hospitalised. It also highlights a specific opportunity for improvement in patient experience and potentially medical outcomes. Additionally, infrastructure that supported best care for people with obesity clearly positively impacted on their lived experience of hospital admission. Previous literature suggests that the physical environment in healthcare settings should allow for a range of body types [29]. Without provision of accessible and supportive built environments, people living with obesity are unable to fully participate in care and may choose (as one participant did) to disengage from treatment completely. The design of hospital environment must therefore consider adequate circulation space, access options and the equipment to accommodate people with obesity in future hospital builds. Retrofitting existing infrastructure is another option but is more costly and often results in a less successful design outcome than universal design from the beginning [30].

Unsurprisingly, the theme of interpersonal interactions was also identified and highlighted negative experiences during hospitalisation. The critical influence of communication on patient experience was repeatedly highlighted as important by participants, who perceived a lack of empathy and feeling ignored. This builds on previous literature in primary care settings, which describe the negative experiences of people living with obesity accessing, or trying to access, healthcare [3]. These previous studies detail a lack of respect and compassion and use of inappropriate language by health care providers. Obesity is a stigmatised and gendered disease which can further impact the relationship between patients and healthcare professionals via implicit or explicit discrimination that impedes patient engagement with treatment [31]. It has been shown that obesity stigma and weight bias is endemic in healthcare settings [32]. Although negative interpersonal interactions described by our participants were not always specifically related to obesity, some data provided evidence of obesity stigma in the hospital setting. In addition, positive interpersonal interactions help people overcome difficult moments in stressful situations such as healthcare [33]. Previous research describes “moments of care” as short-lived, prosocial interpersonal interactions between healthcare consumers and medical staff which significantly contribute to improved experience [33]. This suggests the patient experience may be enhanced by generic cultural improvement which could include small and specific behaviour change such as positive facial expressions and introducing oneself by name. A focus on improving the quality of interpersonal interactions in the hospital setting, particularly though role modelling may assist in reducing discrimination. Healthcare professionals can be role models for tackling discrimination against people with obesity [34]. Teamwork is also critical in this patient cohort that often require specialised equipment and devices, a tailored ergonomic approach and multiple staff to meet care needs [26]. Therefore, supporting staff to develop skills to meaningfully engage with people with obesity may positively impact patient experience.

Obesity is known to be associated with a significant psychosocial burden [35]. This study demonstrated that hospitalisation had a substantial negative impact on emotional and mental wellbeing for people living with obesity. People reported feeling like a burden to staff who were providing care and identified stress related to delays in care delivery. Research has shown that staff perceive inpatients with obesity as passive participants in their care [36], however other studies have shown that people with obesity have a willingness to contribute to care provision that is often overlooked [37]. Of note, people living with obesity often have self-developed solutions to manage self-care that could be well utilised in the hospital setting [38]. Engagement of the patient is critical for patient centred care and should be prioritised by for healthcare professionals. This also presents a strategy to address the identified negative psychosocial impact. Some participants also identified that hospital admission led to self-reflection around weight gain and the subsequent impact on health and wellbeing. They identified their admission was an opportunity to address weight loss and those that did receive support reported this as a positive experience. However, it is not known whether all participants received weight loss support. It is known that healthcare professionals can feel unprepared to provide support with weight loss [34]. This research provides further support for previous suggestions to provide healthcare professionals with specific education to both reduce weight bias and obesity stigma and enable them to support patients in the treatment of obesity and its associated complexities [37]. If staff can build trust through good communication and rapport with people living with obesity when they are in hospital, we may be able to better help engage from a psychosocial perspective to help increase capacity for self-management of their weight. Further research into design of training models to achieve effective health professional education is required.

This study provides a robust investigation of this topic not otherwise represented in the current literature, using a rigorous methodological approach. The sample is representative of hospitalised people with obesity, and includes diverse genders, ages, and reasons for admission. Most participants were morbidly obese with a BMI of greater than or equal to 40, however BMI could not be calculated for two participants due to unknown height. While recruitment targeted acutely admitted inpatients, their experiences included aspects of their entire patient journey, including acute and subacute settings, and post-hospital discharge services. The participants were recruited from a hospital setting with limited specifically designed rooms for people with obesity, and significant infrastructure differences from other sites within the healthcare service. Despite the limitation of being restricted to one public health service, this study therefore included experiences of contrasting settings. The pre-existing initiatives around care for people with obesity at the organisation provided an avenue for recruitment and may also be a platform to implement changes suggested to improve patient experience and ultimately care outcomes. Interviews were guided by a specially designed interview schedule informed by previous research. As such, participant responses and themes identified are likely to align with existing evidence, however this may have restricted the breadth of information gathered.

## Conclusion

This study explored the lived experience of people with obesity admitted to a metropolitan public healthcare service. Findings demonstrate that meeting physical care needs, enhancing interpersonal interactions and understanding and addressing psychosocial impact most significantly influence patient experience. Timely provision of equipment to meet the specialised needs of these patients and consideration of the design of infrastructure was most important, hence should be a key consideration in the redesign of models of care and infrastructure. An emphasis on the basic principles of care provision, coupled with improved understanding of specific needs for people with obesity are also suggested. Role modelling to address obesity stigma and weight bias and enabling healthcare professionals to better support weight loss are likely to positively influence patient experience. The use of consumer engagement strategies would prove beneficial to the development of obesity specific models of care, to ensure better experiences and outcomes for all patients with obesity.

## Supplementary Information

**Additional file 1.**

## Data Availability

The datasets used and/or analysed during the current study available from the corresponding author on reasonable request.
