# Supplementary material for: The lived experience of patients with obesity at a metropolitan public health setting
Source: BMC Health Serv Res. 2022 Dec 16;22:1530. doi: 10.1186/s12913-022-08928-w (PMC9756509; doi:10.1186/s12913-022-08928-w)
Supplement: Supplementary file 1 — Additional file 1. [file 12913_2022_8928_MOESM1_ESM.docx]

**Appendices:**

**Appendix A: Interview Questions:**

**Introduction:** including informed consent, the sensitive nature of the interview, risk management plan and purpose of the interview (including duration and expected outcomes)

## Questions: The following questions are to be used as a guide when conducting the interview. The interview contains five parts: ***A) General Experience, B) Equipment, C) Staff Attitudes and D) Patient Self-Perception, E) Additional Comments.***

1. *General Experience:*

- Overall how would you describe your experience as an inpatient at The Sunshine Hospital? ( follow-up: What made it a good experience? What made it a bad experience? What could have made that experience better?)
- Can you tell me in what way do you think your size may have impacted your admission?
- Do you that you experienced additional difficulties due to your weight?

1. *Equipment:*

- Can you tell me about your experience as an inpatient regarding the supply and use of equipment? (Including timing and how it was delivered to you eg. Staff attitudes)
- In what way did the equipment (eg. bariatric bed, hoist, chair, commode, blood pressure cuff etc) meet or not meet your needs?
- How could the equipment at the hospital be improved to better meet your needs (eg. Were you made to feel that obtaining certain equipment was difficult?)

1. *Staff Attitudes:*

- Did you feel that the staff listened to you and understood what was important to you? (Whether ‘yes’ or ‘no’ Can you give an example?)
- What was your experience of nursing staff; medical staff; allied health? (Follow up: How might these interactions have been improved or what did you find helpful about these interactions?)

1. *Self Perception:*

- Did you feel judged by staff members during your admission due to your weight (provide examples)?
- When thinking about your size what was the most challenging part of your admission?
- Did you feel you were treated differently because of your size? If yes, how so? How did this impact your experience / mood / care / motivation?

1. *Additional Comments:*

- What would you like to be different about your future admissions/ or experiences at Western Health?
- Please provide any other comments or ideas that would assist with improving the way we care for out bariatric inpatients.
